# Inflammatory Biomarkers in Postural Orthostatic Tachycardia Syndrome with Elevated G-Protein-Coupled Receptor Autoantibodies

**DOI:** 10.3390/jcm10040623

**Published:** 2021-02-06

**Authors:** William T. Gunning, Stanislaw M. Stepkowski, Paula M. Kramer, Beverly L. Karabin, Blair P. Grubb

**Affiliations:** 1 Department of Pathology, University of Toledo, Toledo, OH 43614, USA; paula.kramer2@utoledo.edu; 2 Department of Medical Microbiology and Immunology, University of Toledo, Toledo, OH 43614, USA; Stanislaw.Stepkowski@utoledo.edu; 3 Department of Medicine, University of Toledo, Toledo, OH 43614, USA; beverly.karabin@utoledo.edu (B.L.K.); blair.grubb@utoledo.edu (B.P.G.)

**Keywords:** POTS, autoimmune, hypotension, tachycardia, antibody, cytokine, inflammation, syncope, platelet, storage pool deficiency

## Abstract

A growing body of evidence suggests that postural orthostatic tachycardia syndrome (POTS) may be an autoimmune disorder. We have reported in a previous manuscript that 89% of POTS patients (*n* = 55) had elevations in G-protein-coupled adrenergic A1 receptor autoantibodies and 53% had elevations in muscarinic acetylcholine M4 receptor autoantibodies, as assessed by ELISA. Patients with autoimmune disorders have been reported with a variety of elevated cytokines and cytokines (such as rheumatoid arthritis); thus, we evaluated a limited number of cytokines/chemokines in POTS patients with elevated adrenergic and muscarinic receptor autoantibodies. We utilized the plasma of 34 patients from a previous study; all of the patients (100%) had autoantibodies against the A1 adrenergic receptor and 55.9% (19/34) had autoantibodies against the M4 muscarinic acetylcholine receptor. In particular, the plasma cytokine/chemokine levels were measured as biomarkers of inflammation by Quantibody^®^ technology (Raybiotech, Peachtree Corners, GA, USA). We also evaluated the platelet dense granule numbers, as these patients frequently complain of symptoms related to platelet dysfunction. Patients were predominantly young females who displayed a multitude of co-morbidities but generally reported viral-like symptoms preceding episodes of syncope. Eighty five percent (29/34) had platelet storage pool deficiency. Patients had elevations in five of ten cytokine/chemokines biomarkers (IL1β, IL21, TNFα, INFγ, and CD30), whereas two biomarkers had decreased levels (CD40L and RANTES). Our observations demonstrate that POTS patients known to have autoantibodies against the G-protein-coupled adrenergic A1 receptor have abnormal plasma concentrations of inflammatory cytokines.

## 1. Introduction

There is a growing body of evidence suggesting that postural orthostatic tachycardia syndrome (POTS) might be an autoimmune disease. The syndrome was first described by Schondorf and Low in 1993 and included a heterogeneous group of similar clinical physiological presentations [1,2]. Postural orthostatic tachycardia syndrome affects as many as 3 million people in the United States [3], predominantly young women of child bearing age [4,5]. The etiology of the disorder remains unknown. Common clinical symptoms identified in affected patients include light-headedness, dizziness, and fainting episodes; a racing heart; chest pain; abdominal pain and/or nausea; sleep problems; headaches; and cognitive issues. Many of these symptoms are confounders, implicating a variety of potential etiologies of POTS [6,7,8]. A general consensus for the diagnosis of the disorder requires the presence of chronic orthostatic intolerance associated with an increased heart rate of ≥30 beats per minute (BPM) from the supine or sitting basal rate or a rate that exceeds 120 BPM when standing or by an upright tilt test that occurs within 10 min [9,10]. The disorder may be extremely debilitating and the patient with undiagnosed POTS is often referred to a psychiatric service (77% prior to diagnosis) according to a recent publication of data obtained from an international survey of POTS patients [11].

One of the first reports of elevated autoantibodies in dysautonomia was reported by Vernino and co-workers, who proposed that ganglionic acetylcholine receptor (AChR) autoimmunity may cause the condition [12]. A series of subsequent publications by this group led them to suggest that a substantial percentage of POTS cases might be autoimmune [13,14,15,16]. More recently, autoantibodies against G-protein-coupled receptor subtypes have been reported to be elevated in POTS [17,18,19,20,21]. There are also a number of reports of POTS developing subsequent to human papilloma virus immunization [22,23,24], implicating a potential role of inflammation and/or molecular mimicking as etiologies for the development of POTS. There is evidence that predisposing viral infections, celiac disease, and thyroiditis, all of which have a significant immune response, may be associated with POTS [6,10,25]. Other diseases that have been associated with POTS that have significant inflammation include sarcoidosis, alcoholism, Lupus, Sjogren’s Syndrome, heavy metal intoxication, and following chemotherapy [8,26]. In essence, if POTS is an autoimmune disorder as defined by the presence of autoantibodies, it could also be considered an inflammatory disorder.

In a previous study, we also reported a significant number of POTS patients exhibiting a number of symptoms that could be related to platelet dysfunction [7]. Symptoms included easy bruising, epistaxis, heavy menstrual bleeding, and a family history of bleeding; 81% (147/181) of our patients were found to have a platelet delta granule storage pool deficiency (δ-SPD). Platelet δ-SPD is an autosomal dominant disorder but may also be an acquired condition. It appears to be acquired in settings such as in lupus, rheumatoid arthritis, uremia, and myelodysplastic disease [27,28]. There is empirical data suggesting that chronic inflammation may induce acquired δ-SPD. Platelet δ-SPD is also common in Ehlers Danlos syndrome, a comorbidity we have identified in more than a third of our POTS patients. The identification of platelet δ-SPD as a comorbidity in both POTS and EDS is novel and might explain the bleeding symptoms in these patients. It is unknown whether δ-SPD is inherited or acquired in these syndromes.

With evidence that POTS may be an autoimmune disorder, inflammation might be an underlying issue to consider as a mechanism for the development of POTS. Platelets play an essential role in hemostasis but have been recently reported to be essential mediators of inflammation, especially of the innate immune system [29,30]. Could the δ-SPD we have found in a majority of POTS patients be acquired due to an autoimmune process? Could the platelet play a role in potential autoimmunity in POTS? Is this related to an innate immune system activation?

As mentioned above, the platelet is now considered an integral component of the innate immune system. Therefore, it might be possible that an inflammatory condition might also stimulate the platelet degranulation of both alpha and dense granules. This study was intended to explore some biochemical known to be stored, secreted, or expressed or to affect platelets and suggested to mediate inflammation; platelets contain hundreds of cytokines and chemokines, most contained in alpha granules [29,30].

The purpose of this study was to investigate some biomarkers of inflammation, known to be related to platelet activation, in archived plasma from POTS patients that had been evaluated for G-protein-coupled adrenergic and muscarinic cholinergic receptor autoantibodies [18]. Our hypothesis was that patients with G-protein-coupled adrenergic and muscarinic cholinergic receptor autoantibodies would have elevations of cytokines/chemokines, indicating platelet activation or evidence of an inflammatory process, to explain the platelet δ-SPD observed in POTS.

## 2. Materials and Methods

The authors declare that all supporting data are available within the article and its online Supplementary Files. Our descriptive study was approved by the Institutional Review Board of The University of Toledo Medical Center.

### 2.1. Patients

All the patients had histories of orthostatic intolerance manifested by orthostatic tachycardia, weakness, light-headedness, fatigue, and near syncope for at least 6 months or longer and were diagnosed with primary POTS in our Syncope and Autonomic Disorders Clinic. The diagnosis was based upon clinical history, physical examination, and head-upright tilt table analysis in the fasting state. Blood chemistry analysis and thyroid profile analysis were included during diagnostic workups. Patients were excluded if they (1) were on anti-cholinergic, antidepressant, chronic antihypertensive, or diuretic medications; (2) had multisystem disease of any etiology or a diabetic neuropathy; or (3) were immobile for prolonged periods.

**Figure 1:**
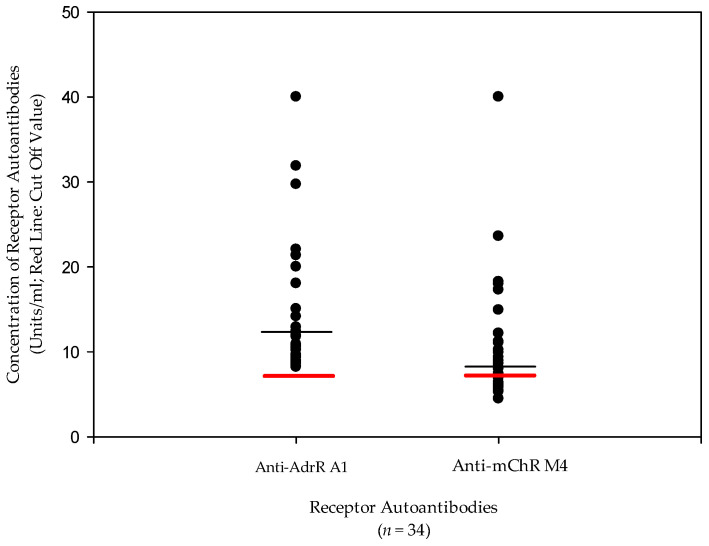
Concentration of adrenergic A1 and muscarinic cholinergic M4 autoantibodies in patients with postural orthostatic tachycardia syndrome. All the subjects included in this study had elevated adrenergic receptor A1 autoantibodies, whereas 19/34 (55.9%) had elevations of muscarinic cholinergic M4 autoantibodies (Pearson’ correlation value *r* = 0.792).

All the patients included in the study (34) were previously reported to have elevations of autoantibodies against the Alpha 1-(A1) adrenergic receptor (Adr-R) and the Muscarinic 4 (M4) anti-muscarinic cholinergic receptor (mAChR) [18]. These autoantibodies had a correlation coefficient of *r* = 0.792. Not all of the patients included in our evaluation of nine different autoantibodies (55) were included in this study due to limitations of available PRP to quantify inflammatory biomarkers. Specifically, we did not have the plasma of normal subjects nor POTS patients who did not have elevations of adrenergic receptor antibodies in our assessment of adrenergic and muscarinic receptor autoantibodies available for this study. In contrast to our previous report that found the most significant elevation of the alpha 1 adrenergic receptor (AdrR A1) autoantibody (ab) in 89% (49/55) of POTS patients, all thirty-four patients in this study had elevated AdrR A1 Abs (Figure 1). Patient symptoms and the results of all adrenergic and muscarinic receptor autoantibody assays for these 34 subjects are available in Supplementary Table S1 and Supplementary Figures S1 and S2.

**Figure 2:**
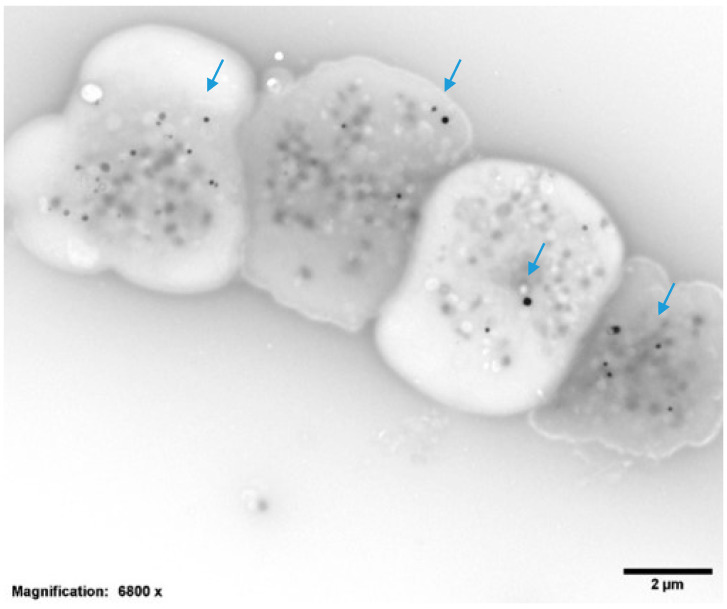
Representative transmission electron microscopy image of four (4) whole-mounted and air-dried platelets. Dense granules appear as opaque round bodies (arrows; normal = 4–6/platelet; range 0–30/platelet), whereas the ill-defined gray bodies are alpha granules (50–80/platelet).

As above, we have previously reported that many POTS patients report symptoms of easy bruising and frequent nosebleeds, and, for women, heavy menstrual bleeding [7]. These symptoms suggest potential platelet dysfunction. We utilized two (2) assessment tools to determine whether the bleeding symptoms were of relative significance. The first bleeding assessment tool was a means to objectively quantify menstrual bleeding; a pictorial representation of blood absorbed by tampons or pads can be scored by determining the number of days of menses and the numbers and degrees of blood absorbed on tampons and pads [31]. We also used a slightly modified bleeding assessment tool (BAT), first described as the Vicenza Bleeding Questionnaire [32] and further developed by an International Society on Thrombosis and Haemostasis (ITSH) committee for a screening tool to assess patients with unexplained bleeding [33]. Our modifications were minor (i.e., “I have…” rather than “do you have…”) so that the patient could check off bleeding symptoms rather than requiring the BAT to be administered by a health professional. The ITSH BAT is currently the preferred tool to determine the relative bleeding significance of suspected platelet dysfunction for the screening of patients with unexplained bleeding symptoms. With a prevalence of symptoms related to platelet dysfunction, we evaluated the mean platelet delta granule number for the patients included in this study. All patient blood samples were aliquoted to obtain a complete blood cell count (CBC) and to prepare samples for the evaluation of the platelet dense (delta) granule number (Figure 2).

### 2.2. Platelet Preparations for Electron Microscopy

Platelet rich plasma (PRP) was obtained from whole blood by centrifugation at room temperature for 15 min at 200× *g*. Electron microscopy coated copper grids used for platelet support were washed with deionized water following PRP incubation and air-dried. An FEI Tecnai G2 Spirit BioTwin transmission electron microscope (TEM, Hillsboro, OR, USA) was used to determine an average number of DG/PL. Previous studies from this laboratory have established a normal of 4.64 ± 0.11 (Mean ± 1 SE DG/PL), consistent with the established literature [34,35].

### 2.3. ELISA Sample Preparation

Subsequent to the preparation of the whole-mounted platelets for EM, the remaining samples were centrifuged to obtain the platelet poor plasma (PPP) and stored frozen. Autoantibodies were evaluated using ELISA kits that were purchased from CellTrend GmbH (Luckenwalde, Germany). To detect autoantibodies against the A1 adrenergic receptor epitope, a CellTrend ELISA kit number 12,400 was used, and for the M4 muscarinic cholinergic receptor epitope kit number 15,400 was used. All the procedures followed the manufacturer’s instructions for each kit and included standards and controls for incubation with test samples. The cut-off values utilized for the determination of elevated antibody titers were established by the manufacturer for both kits. Both kits have been validated by the manufacturer and used successfully in a number of published studies, including ours [36,37]. In general, both assays utilized 100 µL of a sample, standard, or control for 2 h of incubation at 4 °C and this was followed by a wash step, a 1 h incubation at room temperature for the detection of antibodies, another wash step, a substrate incubation for 20–30 min at room temperature, and finally the addition of a stop solution prior to spectrophotometry.

### 2.4. Inflammation Biomarker Preparations

Custom Quantibody^®^ multiplex ELISA array slides purchased from RayBiotech, Inc. (Cat. QAA-CUST, Peachtree Corners, GA, USA) were utilized to assess 10 substances that have been associated with inflammation and platelet activation [29,38,39,40].

**Figure 3:**
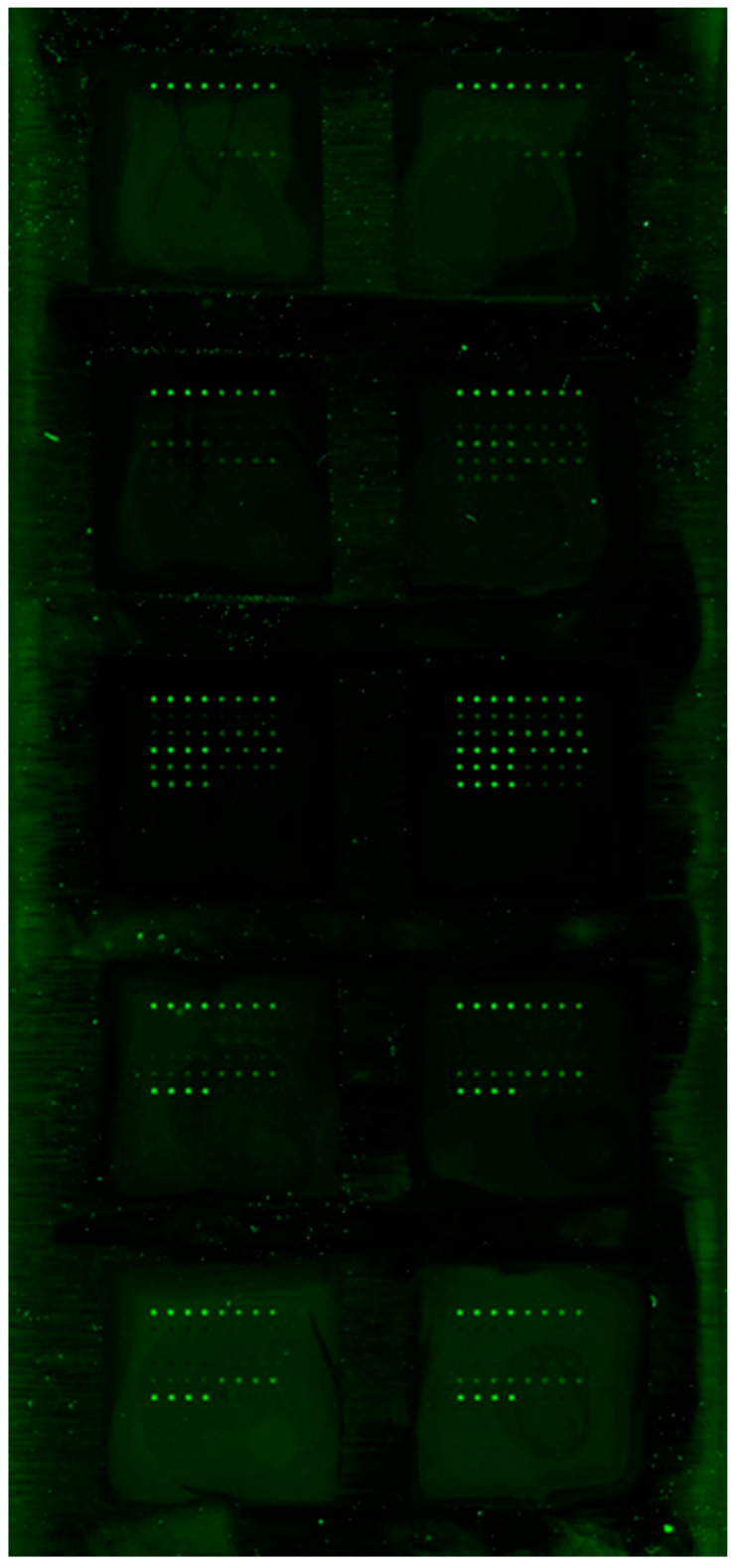
Representative Quantibody^®^ multiplex ELISA array slide analyzed by RayBiotech, Inc. to provide quantitative concentrations of the ten cytokines/chemokines for each of the 34 postural orthostatic tachycardia syndrome (POTS) patients evaluated in this study.

The multiplex ELISA system allowed for the simultaneous quantification of cytokines/chemokines. Briefly, antibodies specific to IL-1β, IL-10, IL-21, TNFα, INFγ, CD30, sCD40L (CD154), RANTES (Regulated on Activation, Normal T Expressed and Secreted, CCL5), p-selectin, and MCP-1 (chemokine ligand 2/CCL2) were used by the company to create subarrays in quadruplicate and included a protein standard mix. These substances were selected based upon reports in the literature that identified they were either stored, processed, secreted, or affected platelets. The system uses matched pairs of antibodies for capture similar to a standard sandwich-based ELISA and quantitation made by comparison with the protein concentration from a standard curve. For each patient, 50 µL of PRP was placed in an incubation chamber affixed to a glass slide that was gently rocked for 2 h and subsequently processed as recommended by the manufacturer. All the samples were sent to RayBiotech for scanning and the quantification of cytokine concentrations. An example of an assayed Quantibody^®^ slide is represented in Figure 3.

### 2.5. Statistical Methods

Unless otherwise stated, data are presented as mean +1 standard error of the mean (SE). Pearson’s correlation test was used compare our clinical symptom severity scores with the cytokine concentrations. All the statistical analyses were performed and graphed using SigmaPlot^®^ (SSPS, Inc., Chicago, IL, USA).

## 3. Results

### 3.1. Bleeding Assessments

Easy bruising was described by most POTS patients (23/34; 67.6%); this symptom is common for patients with platelet dysfunction disorders. Other symptoms that suggest platelet dysfunction included epistaxis, heavy menstrual bleeding (HMB), gum bleeding with tooth brushing, and excessive bleeding following surgical procedures (Table 1; Supplementary Table S1). We obtained eight pictorial menses assessments and, of these, four (50%) had significant HMB ranging from scores of 235 to 485 (HMB defined as pictorial score >185) (Table 1) [31]. A BAT was recorded for twenty-one of the subjects and demonstrated an elevated mean bleeding score of 6.9 (normal ≤3 for males and ≤5 for females) and a range of scores from −2 to 27; two of the women had scores greater than 20. A BAT was obtained for one of the two men and was found to have no bleeding symptoms with a −1 score; eight women (8/20: 40%) had normal BAT scores.

### 3.2. Complete Blood Cell Count and Platelet Storage Pool

Twenty-five POTS patients were found to be anemic on complete blood cell count (CBC), including one of the two men. None were thrombocytopenic, however twelve had low mean platelet volumes (Supplementary Table S2). An analysis of the platelet whole mounts found an average of 3.04 ± 0.9 DG/PL (normal = 4–6 DG/PL) for the entire group. Twenty-nine patients (29/34; 85.3%) were found to be platelet delta granule storage pool-deficient (δ-SPD), which is similar to a previous study of ours that found that 81% of 181 POTS patients had δ-SPD. There was a statistical correlation with hemoglobin and hematocrit (*r* = 0.096, *p* = 0.009), but not with other hematologic factors measured, including the platelet dense granule number.

### 3.3. Biomarkers of Inflammation

Of the three interleukins we chose to study, IL1β and IL21 were significantly elevated compared with normal values, whereas IL10 was within normal limits (Table 2). Tumor necrosis factor alpha (TNFα), interferon gamma (INFγ), and CD30 were also elevated compared to normal values (Table 2). P-selectin and monocyte chemoattractant protein-1 (MCP-1) were within normal limits, but soluble CD40 ligand (sCD40L/CD154) and RANTES (CCL5) were found to be at significantly lower values than normal (Table 2). There were no correlations of cytokines/chemokines with autoantibodies (Table 3), but many cytokine/chemokine correlations were identified using Pearson’s correlation coefficients, highlighted in red font in Table 4.

In this study, we evaluated the hypothesis that a cytokine or chemokine might be identified related to platelet activation to explain the observation that POTS patients seem to have a significant association with δ-SPD. We found that the majority of our POTS patients had platelet δ-SPD, consistent with findings we have previously reported [7]. We also identified significant elevations of IL1β, IL21, TNFα, INFγ, and CD30 in the plasma of POTS patients with elevated autoantibodies to at least the A1 adrenergic receptor. These five biomarkers of inflammation are all mediators of the innate immune system. Thus, POTS patients appear to have evidence of an ongoing inflammatory process in addition to the previously described autoimmune process.

A recent review article by Mantovani et al. (2019) describes that the Interleukin-1 family is composed of 11 soluble molecules and 10 receptors, divided into subgroups based upon molecular sequence and activity including agonist activity, receptor antagonists, and an anti-inflammatory cytokine [41]. They conclude that IL-1 represents a paradigm for inflammation and immunity as a metanarrative of 21st century medicine; this could also apply to our understanding of POTS, with reported evidence of autoantibodies as a potential etiology of the disorder.

Leukocytes, including monocytes, macrophages, neutrophils, and dendritic and endothelial cells, are the major source of IL-1β, produced by the caspase cleavage of a pre-protein [42]. Tunjungputri, et al. (2018) found increased plasma levels of IL-1β that were positively associated with platelet numbers, p-selectin expression, and several platelet single-nucleotide polymorphisms [30]. IL-1β is an essential cytokine in innate immunity as one of the first signals in plasma in response to infection [29,42]. IL-1β has been reported to be highly involved with autoimmune and autoinflammatory diseases; the pathogenesis of autoimmune disease involves genetic susceptibility [43]. Classic autoimmune disorders are characterized by the presence of autoantibodies and autoantigen-specific T cells [44]. The symptoms of autoimmune disease and autoinflammatory disorders overlap significantly. Autoinflammatory diseases are characterized by innate immunity abnormalities, usually without infections and without autoantibodies [45,46,47]. Autoimmune disorders are propelled by type I interferon, whereas autoinflammation is distinguished by elevations of inflammasome-induced IL-1β and IL-18; IL-1β and type I IFN counter-regulate one another and interfere with adaptive immune responses [45]. IL-1β mediates immunity for both innate and adaptive responses; it promotes innate immunity via the recruitment of inflammatory cells, whereas it enhances T cell differentiation for adaptive immunity [48]. Since we have identified elevations of both autoantibodies against adrenergic and muscarinic receptors in our study group, the approximate 30-fold elevation of IL-1β (Table 3) in these patients supports published data that suggest POTS is an autoimmune disorder, presumably via the persistent activation of T cells. A schematic diagram of the cytokine/chemokines described in this manuscript and interactions with immune cells is available as Supplementary Figure S3.

The IL-21 levels were elevated approximately 10-fold (Table 4) in these patients. IL-21 is produced by CD4 + T cells, natural killer T cells (NKT), and follicular helper cells and induces B cell proliferation and differentiation into plasma cells [42]. IL-21 can also be immunosuppressive because of its ability to induce IL-10 [49]. Elevations of IL-21 have been reported in the pathogenesis of some autoimmune diseases, including celiac disease (CD), rheumatoid arthritis (RA), and systemic lupus erythematous (SLE) [50,51].

Tumor necrosis factor alpha (TNFα) was approximately 100-fold elevated in our patients (Table 4); it is an extremely important signaling cytokine of the innate immune system that is involved in acute phase reactions and produced primarily by activated macrophages [42]. It is also produced by T helper and NKT cells in response to IL-1; has antiviral properties [52]; and is implicated in major depression, which is a common comorbidity in POTS [53]. It has recently been reported that TNFα induces the inflammasome-independent production of IL-1β, causing autoimmunity [48]. With elevations of IL-1β, IL-21, and TNFα, there is a strong implication that the development of autoantibodies in our patients may be T-cell-mediated.

Unfortunately, we did not measure the plasma levels of interferons alpha and beta (INFα/β, type I INF), which are produced by monocytes and fibroblasts and considered “non-immune interferons” [54]. We did identify 40-fold elevations (Table 4) in INF gamma (INFγ, type II INF) that is produced by cytotoxic and helper T cells and can activate macrophages and NKT cells, both known to be elevated in viral infections [55]. We chose INFγ, a type II interferon, specifically thinking of a potential viral etiology for POTS, and this is a significant limitation of our study. Type I interferons have become increasingly recognized as important cytokines in autoimmune diseases such as rheumatoid arthritis (RA) and systemic lupus erythematous (SLE) [56,57].

We included measurements of CD30, a transmembrane receptor expressed by activated T and B cells; it is part of the TNF receptor superfamily [58]. CD30 expression has been reported in many autoimmune diseases, including RA [59], and is thought to have regulatory effects to inhibit autoimmunity [60]; it is also elevated in viral infections [58]. The 20-fold elevation (Table 4) in our POTS patients might be regulatory in an attempt to inhibit the autoimmunity, or it might be related to a recent or chronic viral infection. This implicates potential pathways for POTS, similar to those in the etiology of RA.

Two of the inflammation biomarkers we assayed had significantly lower plasma levels than normal. CD40 ligand (CD40L) is a transmembrane molecule anchored in activated T cell and platelet membranes [61]. It can be cleaved into soluble CD40L (sCD40L), and both are important mediators of inflammation and immunity. Elevations of sCD40L have been associated with morbid obesity; cardiovascular problems; and autoimmune disorders, including RA and SLE [62,63]. We were unable to identify a specific disorder associated with decreased plasma levels via a thorough literature review. It might be possible that platelet δ-SPD is an acquired condition related to chronic inflammation, and the platelets cannot synthesize nor store CD40L, resulting in the decrease in the plasma.

We also found that RANTES (CCL5) was significantly lower than normal values; this was unexpected. RANTES is a strong biomarker of platelet activation and a potent mediator of inflammation. It is reported to be elevated in numerous diseases. Our results of decreased RANTES in POTS patients do not support our hypothesis that platelets are activated in POTS. RANTES is also secreted by activated monocytes, but not to the extent of activated platelets. A question remains regarding the decreased RANTES in our patients’ plasma; our results suggest that neither platelets nor monocytes are activated, therefore RANTES is not secreted, which would elevate the substance. This result is unexpected and of unknown significance.

The limitations of our study include many of the deficiencies of a descriptive, proof of concept study. These include the lack of a case control study, a low number of subjects (34 in this report), and an incomplete number of cytokines/chemokines utilized. We did not include assessments of IL-6, IL-8, IL-17, IL-18, CD40, and type I IFNα/β. These will be included in a future case control study to validate our findings. This is not a review in immunology, but we have included discussions of cytokine/chemokine sources and their respective functions in an attempt to rationalize our findings, as five of the inflammatory biomarkers were elevated, three were within normal limits, and two unexpectedly reduced (Table 4). An additional problem of data interpretation includes that there are no established standardized normal ranges and cut-off values for the cytokines/chemokines that we assayed. As above, a planned case control study will allow us to establish our own cut-off values. Our Quantibody^®^ multiplex ELISA assay provided a quality control standardized curve to determine concentrations with the lower limit of detection established by RayBiotech. We reviewed as many manuscripts as we could identify with control group biomarker ranges and scoured ranges from most of the large reference laboratory websites to find a reasonable normal range of expected biomarker concentrations we could use for our results. Finally, we did not record the therapeutic drug history and nor was BMI recorded for these patients. All the patients have been de-identified and we cannot review their medical records.

Our patients demonstrated elevations of five biomarkers of inflammation (IL-1β, IL-21, TNFα, INFγ, and CD30) that are known to be elevated in autoimmune disorders such as RA and SLE [64]. The development of autoantibodies in POTS could potentially be due to a dysregulation of an interleukin, such as is seen in the pathogenesis of SLE with elevations of IL-21, TNF, and INFγ, as observed in our patients [65].

Future plans include a comprehensive case control study to confirm our previously reported finding of autoantibodies against both adrenergic and muscarinic receptors in POTS patients. We also plan to confirm the results of this report but will include additional cytokines and chemokines, as mentioned above. Of particular interest is addressing the etiology of δ-SPD, which is likely to be an acquired condition rather than an inherited comorbidity.

The innate immune system, including the platelet, is important in defense against viral infections. Many autoimmune diseases have been hypothesized to evolve from an antecedent viral infection, and there are also a few reports that suggest that vaccinations may, on occasion, induce POTS [66,67]. Other studies have reported that viral infections and molecular mimicry are likely associated with autoimmune diseases [68,69]. A number of recent reports have identified POTS as a sequela of COVID-19 infection [70,71,72]. One might postulate that molecular mimicry might induce antibodies that target adrenergic and cholinergic muscarinic receptors via receptor stoichiometry similar to an unknown antigenic epitope seen in infection and/or inflammation. A significant number of our patients have described Epstein Barr virus infections and gastrointestinal pain that could be related to an enteric viral infection preceding the onset of symptoms and ultimately the development of POTS. It may also be that the gastrointestinal pain is related to the hypomotility of the intestinal tract due to a deficiency of serotonin available to drive contraction of smooth muscle cells. Irritable bowel syndrome (IBS), a common co-morbidity in POTS, is possibly linked to the reduced secretion of serotonin by enterochromaffin cells in the gut. Enterochromaffin cells can be enhanced or attenuated by the secretory products of immune cells, such as CD4 + T cells, and patients with IBS have fewer serotonin-producing cells in the large intestine than normal control subjects [73,74]. The platelet stores 99% of the serotonin outside of the central nervous system in dense granules, and more than 80% of our patients have a deficiency of platelet dense granules. We did not have evidence of platelet activation to explain the δ-SPD; it could be possible that the deficiency is related to low serotonin production in the gut and therefore results in fewer dense granules. Regardless, our hypothesis regarding platelet activation is incorrect.

## 4. Discussion

In conclusion, our study demonstrated that, in a small group of POTS patients, there were elevations of cytokines and chemokines characteristic of an innate immune condition, similar to autoimmune diseases including RA, SLE, psoriasis, systemic sclerosis, multiple sclerosis, and type-1 diabetes [48,75,76]. We believe that the identification of increased levels of pro-inflammatory cytokines IL-1β, IL-21, TNFα, INFα, and TNF receptor (CD30) in POTS patients with autoantibodies against adrenergic and cholinergic muscarinic receptors is highly suggestive of a coexisting inflammatory process that contributes to the disorder and requires further study.

## Figures and Tables

**Table 1:** Characteristics and co-morbidities of patients with postural orthostatic tachycardia syndrome.

Demographic/Symptom	Mean (STD)/Percentage/(N)
N	34
Age	31.1 ± 11.5
Females	94.1% (32/34)
Menses score	202.6 ± 145.3 (8/32), Normal: <185
Bleeding score	6.9 ± 7.9 (21), Normal: <5 for women
Easy bruising	67.6% (23)
Dense Granules per Platelet (DG/PL)	3.04 ± 0.9, Normal = 4–6 DG/PL

**Table 2:** Inflammatory cytokine and chemokine biomarkers in postural orthostatic tachycardia syndrome (POTS) patients with elevated adrenergic and muscarinic autoantibodies.

Cytokine/Chemokine	POTS Patients (*n* = 34) (pg/mL)	Normal (pg/mL)	Major Function
IL 1β	332 ± 100	<10	Regulates cell proliferation
IL 10	16 ± 3.6	<6	Inhibitory to T helper cells
IL 21	1918 ± 410	<200	Controls NK and T cells
TNFα	342 ± 78	<3	Regulates inflammation
INFγ	226 ± 62	<5	Antiviral
CD30	193 ± 59	<10	Regulates cell proliferation
CD40 L	119 ± 11	350–90	Recruits leukocytes
RANTES (CCL5)	995 ± 123	5000–6100	Chemotactic for T cells
P-Selectin	12,540 ± 1094	10,000–130,000	Recruits leukocytes
MCP-1	78 ± 5	65–1025	Recruits monocytes
AdR A1 antibodies	16.6 U/mL	<7 U/mL	Autoantibody
AChR M4 Abs	11.2 U/mL	<7 U/mL	Autoantibody

Red font signifies elevations, black signifies normal levels, and blue font signifies decreased values.

**Table 3:** Correlation between cytokines/chemokines, autoantibodies, and platelet dense granules.

Autoantibodies		IL-1β	IL-10	IL-21	TNFα	INFγ	CD30	CD40L	RANTES	P-Selectin	MCP-1
AdrR A1	*r*	−0.067	0.194	−0.007	0.093	0.086	0.100	−0.170	−0.029	−0.198	−0.132
	*p*	0.705	0.272	0.966	0.602	0.627	0.573	0.338	0.236	0.261	0.458
AChR M4	*r*	0.018	0.281	−0.061	0.254	0.210	0.211	−0.264	−0.139	0.203	0.156
	*p*	0.919	0.107	0.731	0.147	0.238	0.231	0.231	0.131	0.433	0.379
DG	*r*	−0.107	0.101	0.445	−0.025	0.196	0.338	−0.182	−0.634	−0.357	−0.077
	*p*	0.100	0.871	0.883	0.968	0.752	0.578	0.769	0.250	0.555	0.902

AdrR A1: adrenergic receptor A1; AChR M4: acetylcholine receptor M4; DG: platelet dense granule.

**Table 4:** Correlation between cytokines/chemokines.

		IL-1β	IL-10	IL-21	TNFα	INFγ	CD30	CD40L	RANTES	P-Selectin	MCP-1
IL-1β	*r*		0.653	0.520	0.606	0.565	<0.87	0.106	0.866	0.196	0.379
	*p*		<0.001	0.002	<0.001	<0.001	<0.001	0.552	<0.001	0.266	0.027
IL-10	*r*			0.555	0.731	0.694	0.83	0.011	0.218	−0.141	0.093
	*p*			<0.001	<0.001	<0.001	<0.001	0.950	<0.001	0.427	0.601
IL-21	*r*				0.74	0.76	0.626	0.133	−0.019	−0.121	0.242
	*p*				<0.001	<0.001	<0.001	0.453	0.912	0.497	0.168
TNFα	*r*					0.93	0.81	−0.041	0.105	−0.150	0.447
	*p*					<0.001	<0.001	0.817	0.553	0.398	0.008
INFγ	*r*						0.73	0.043	0.073	−0.075	0.388
	*p*						<0.001	0.808	0.680	0.674	0.024
CD30	*r*							0.049	0.215	0.007	0.338
	*p*							0.784	0.222	0.970	0.051
CD40L	*r*								0.367	0.134	−0.011
	*p*								0.033	0.449	0.95
RANTES	*r*									0.231	−0.008
	*p*									0.188	0.962
P-Selectin	*r*										0.076
	*p*										0.671

Pearson’s correlation *r* and *p* values high-lighted as red font indicate significance.

## Data Availability

All the data utilized in this study are included in the manuscript or provided in the supplementary figures and table.

## Supplementary Materials

The following are available online at https://www.mdpi.com/2077-0383/10/4/623/s1, Figure S1: Concentration of adrenergic autoantibodies in patients with postural orthostatic tachycardia syndrome, Figure S2: Concentration of muscarinic cholinergic autoantibodies in patients with postural orthostatic tachycardia syndrome; Figure S3: Schematic diagram of cytokine/chemokine interactions with immune cells, Table S1: Characteristics and co-morbidities of patients with postural orthostatic tachycardia syndrome, Table S2: Complete blood cell count of patients diagnosed with postural orthostatic tachycardia syndrome.
