# Supplementary material for: Inflammatory Biomarkers in Postural Orthostatic Tachycardia Syndrome with Elevated G-Protein-Coupled Receptor Autoantibodies
Source: J Clin Med. 2021 Feb 6;10(4):623. doi: 10.3390/jcm10040623 (PMC7914580; doi:10.3390/jcm10040623)
Supplement: Supplementary file 1 [file jcm-10-00623-s001.zip › Supplemental Figure 3. Schematic Diagram Final.docx]

Figure S3: Schematic Diagram of Cytokine/Chemokine Interactions with Immune Cells.

IL-1β, IL-10, INFγ, RANTES


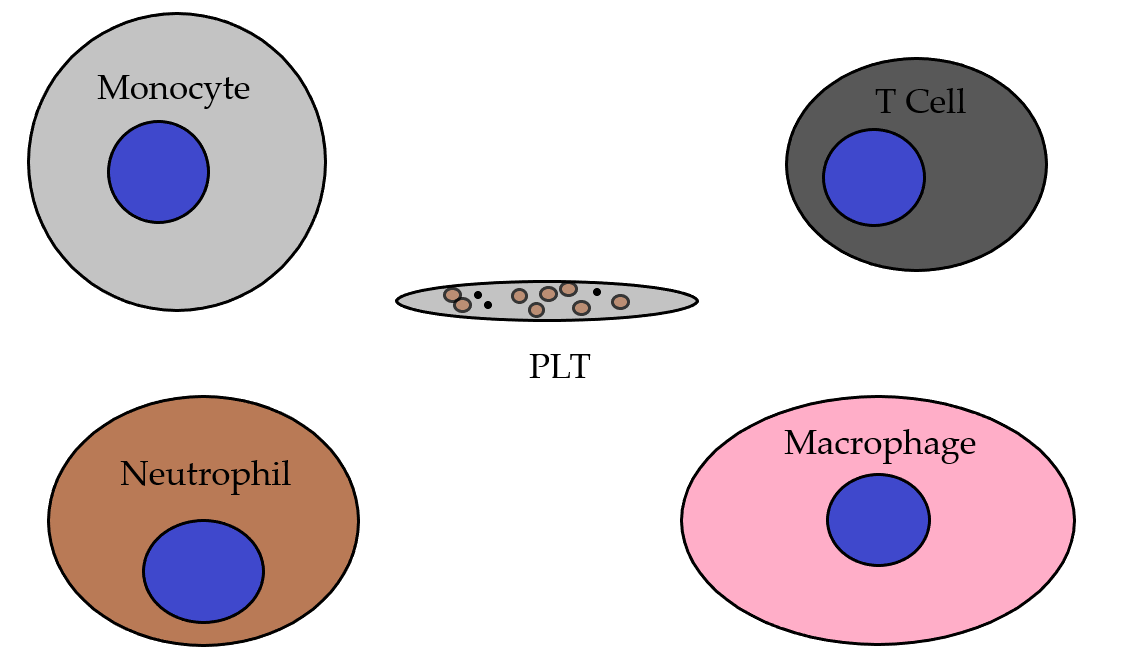


IL-21

IL-1β

IL-10

IL-21

INFγ

TNFα

CD30

TNFα

IL-1β

TNFα

IL-1β, RANTES,

TNFα, CD40L

IL-1β

INFγ

TNFα

IL-1β

IL-10
