# Supplementary material for: Inflammatory Biomarkers in Postural Orthostatic Tachycardia Syndrome with Elevated G-Protein-Coupled Receptor Autoantibodies
Source: J Clin Med. 2021 Feb 6;10(4):623. doi: 10.3390/jcm10040623 (PMC7914580; doi:10.3390/jcm10040623)
Supplement: Supplementary file 1 [file jcm-10-00623-s001.zip › Supplemental Table 1 Other Symptoms.docx]

**Supplemental Table 1. Characteristics and Co-Morbidities of Patients with**

**Postural Orthostatic Tachycardia Syndrome**

**Other Clinical Symptoms**

Dizzy spells 100% (34)

Fatigue 97.1% (33)

Palpitations 82.4% (28)

Arthralgia 76.5% (26)

Nausea 73.5% (25)

Migraine 70.6% (24)

Joint Hyperflexibility 64.7% (22)

Irritable bowel syndrome 61.8% (21)

Cognitive issues 61.8% (21)

Muscle weakness 61.8% (21)

Anemia 61.8% (21)

Shortness of breath 58.8% (20)

Depression 58.8% (20)

Raynaud’s syndrome 55.9% (19)

Anxiety 52.9% (18)

Depression 38.2% (13)

Heavy menstrual bleeding 50.0% (4/8)

Epistaxis 32.4% (11)

Sleep disorders 20.6% (7)

**Mean Number of Symptoms Reported Per POTS Patient**

49.4/103 symptoms recorded
