# Supplementary material for: Inflammatory Biomarkers in Postural Orthostatic Tachycardia Syndrome with Elevated G-Protein-Coupled Receptor Autoantibodies
Source: J Clin Med. 2021 Feb 6;10(4):623. doi: 10.3390/jcm10040623 (PMC7914580; doi:10.3390/jcm10040623)
Supplement: Supplementary file 1 [file jcm-10-00623-s001.zip › Supplemental Table 2. CBC.docx]

**Supplemental Table 2. Complete Blood Cell Count of Patients Diagnosed with Postural Orthostatic Tachycardia Syndrome**

| **Category** | **Value** | **Std. Dev.** | **Normal** | **Unit** |
| --- | --- | --- | --- | --- |
| RBC | 3.8 | 0.3 | 4.2-5.4 | 10^6^/µl |
| WBC | 6.1 | 2.1 | 4.3-10.8 | 10^6^/µl |
| NE % | 58.5 | 8.0 | 45-75 | % |
| LY % | 32.5 | 7.7 | 16-46 | % |
| MO % | 6.3 | 2.5 | 3-7 | % |
| EO % | 2.1 | 1.9 | 1-3 | % |
| BA % | 0.5 | 0.3 | 0-2 | % |
| HGB | 11.2 | 10.9 | 12.1-15.1 | g/dL |
| HCT | 34.4 | 2.6 | 36.1-44.3 | % |
| MCV | 89.9 | 4.1 | 80-100 | fL |
| MCH | 29.3 | 1.6 | 27-32 | pg |
| MCHC | 32.5 | 0.6 | 32-36 | % |
| RDW | 13.6 | 1.9 | 11-15 | % |
| PLT | 224.9 | 50.6 | 100-400 | 10^6^/µl |
| MPV | 7.3 | 0.8 | 7-10 | fL |
